# Abducens nerve palsy secondary to allergic fungal sinusitis

**DOI:** 10.1093/jscr/rjae697

**Published:** 2024-11-16

**Authors:** Majd A Alsaleh, Ali A Almomen

**Affiliations:** Department of Otorhinolaryngology, Aljabr Eye and ENT Hospital, Alhassa 36422, Saudi Arabia; Department of Otorhinolaryngology, King Fahad Specialist Hospital, Dammam 32253, Saudi Arabia

**Keywords:** allergic fungal sinusitis, abducens nerve palsy, paranasal sinuses

## Abstract

Allergic fungal rhinosinusitis (AFRS) stands out as the predominant form of fungal sinusitis, primarily attributable to a hypersensitive response to fungal invasion. AFRS Characterized by symptoms of rhinosinusitis. The expanding mass in the disease leads to bony restructuring and implicating adjacent anatomical structures. AFRS may extend beyond the sinus cavities, leading to compression of nearby structures like the orbit, optic and abducens nerves which leads to many complications such as nerve palsies and proptosis. Diagnosis of AFRS typically necessitates radiographic assessment initially, with histopathological examination serving as the confirmatory modality. The management of AFRS typically entails a multifaceted approach integrating surgical intervention alongside medical therapy. This case report illustrates a distinctive manifestation of abducens nerve palsy secondary to allergic fungal sinusitis which had dramatic improvement and resolution of the diplopia after the Endoscopic sinus surgery. Steroids and nasal saline irrigation have been prescribed post operatively to prevent the recurrence.

## Introduction

Allergic fungal rhinosinusitis (AFRS) represents a unique variant of non-invasive chronic sinusitis originating from a localized allergic reaction to non-invasive fungal proliferation. The manifestations of AFRS resemble those observed in chronic rhinosinusitis (CRS) accompanied by nasal polyps, as all individuals with AFRS exhibit nasal polyposis. Early-stage patients may experience symptoms such as nasal congestion or obstruction, anosmia, and/or postnasal drip [1, 2]. The diagnosis of AFRS is based on a combination of clinical, radiological, microbiological, and histopathological findings, in addition to the Bent and Kuhn criteria which entail hypersensitivity to fungal components, the presence of nasal polyps, specific findings on computed tomography scans, eosinophilic mucus without fungal infiltration, and positive results on fungal staining [1, 3]. Surgical treatment remains the primary treatment modality which associated with a good prognosis [4]. In severe instances, AFRS may extend beyond the sinus cavities, leading to compression of nearby structures like the optic and abducens nerves [2]. This article showed a rare complication of AFRS that present with abducens nerve palsy. It was diagnosed and managed at King Fahad Specialist Hospital-A tertiary care hospital in Dammam, Saudi Arabia.

## Case report

A 39 year old Saudi male patient, known case of allergic fungal sinusitis, underwent Functional Endoscopic Sinus Surgery (FESS) four times, last one at 2021. He was symptoms free for 1 year post FESS. He is known to be Kidney donor since 2016. He is following up with neurology clinic since 2022 for paresthesia and abnormal lower limb sensation, and currently he is on amitriptyline. He is following up with endocrine clinic for hyperprolactinemia and currently he is on cabergoline.

**Figure 1:**
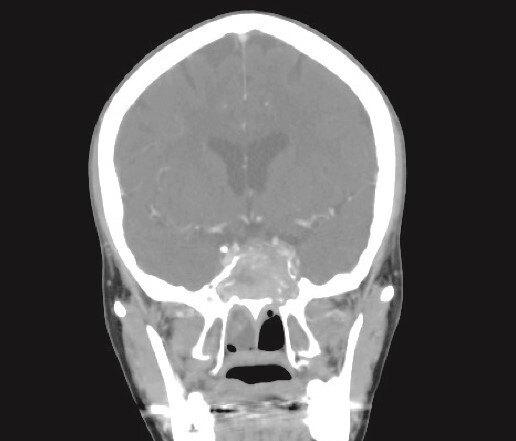
CT brain, coronal reconstruction shows bilateral near total opacification and expansion of the paranasal sinuses and nasal cavity. Invasion of the left cavernous sinus with encasement of cavernous segment of left ICA. Pituitary gland and optic chiasm displaced superiorly.

**Figure 2:**
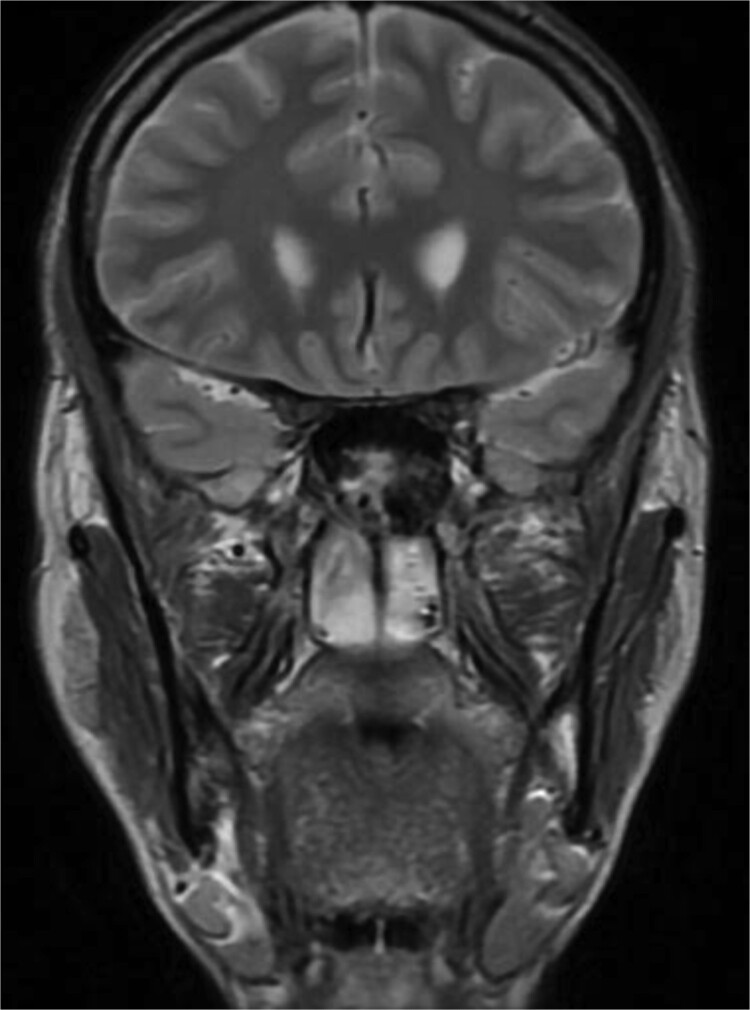
MRI of paranasal sinuses shows the sphenoid sinus is expanded and bulging superiorly into the pituitary fossa causing displacement of pituitary gland and stalk without mass effect upon the optic chiasm. It extends laterally causing effacement of the cavernous sinus.

**Figure 3:**
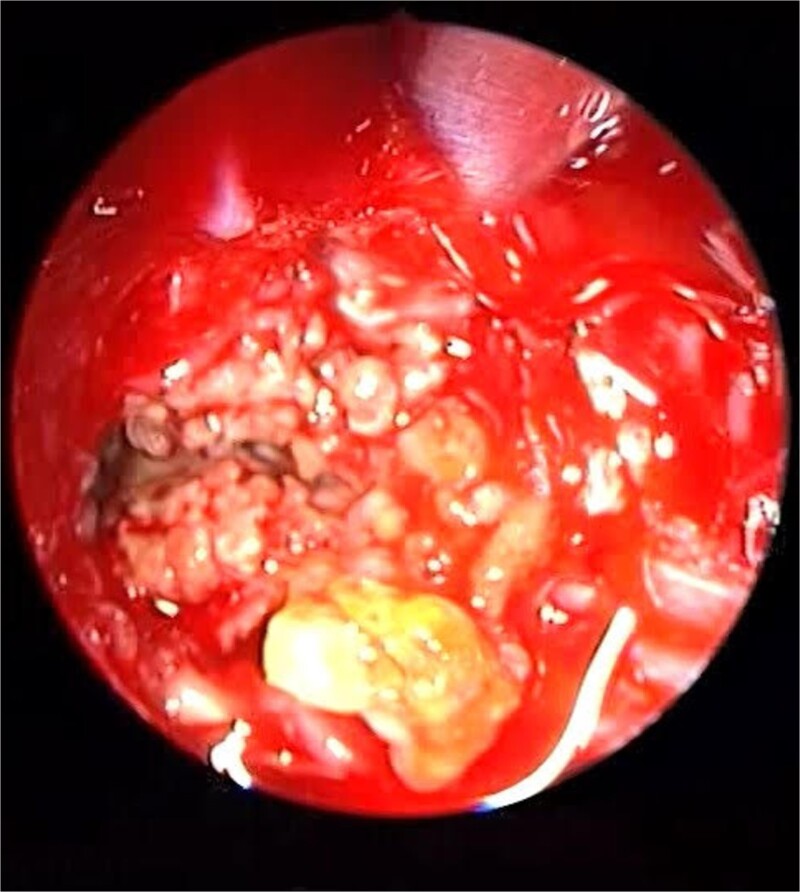
Endoscopic intraoperative view of the sphenoid sinus full of fungal mud and mucin.

**Figure 4:**
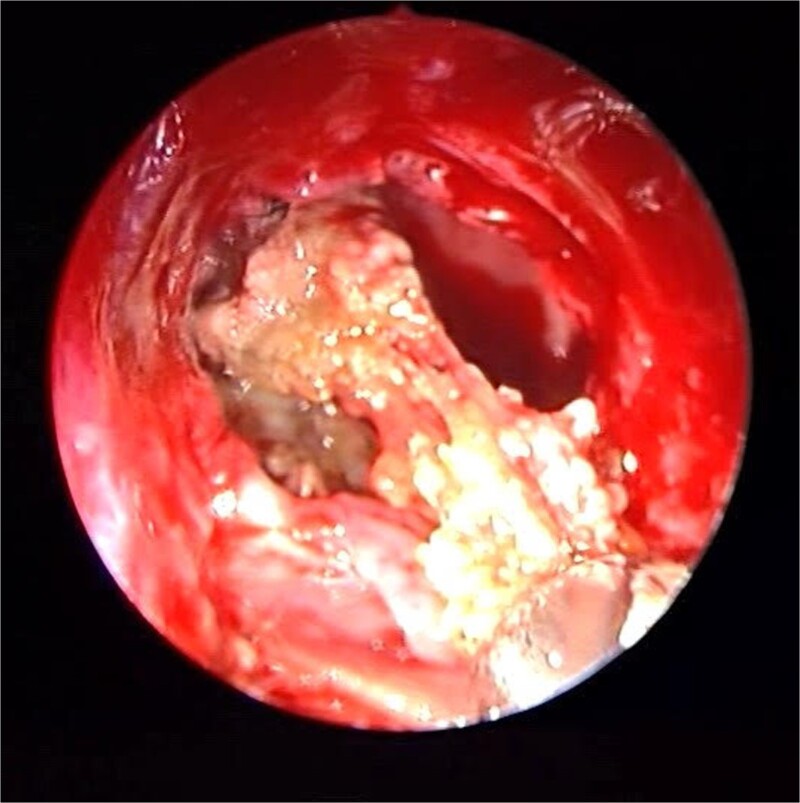
The thick fungal mucin is removed from the sinus.

**Figure 5:**
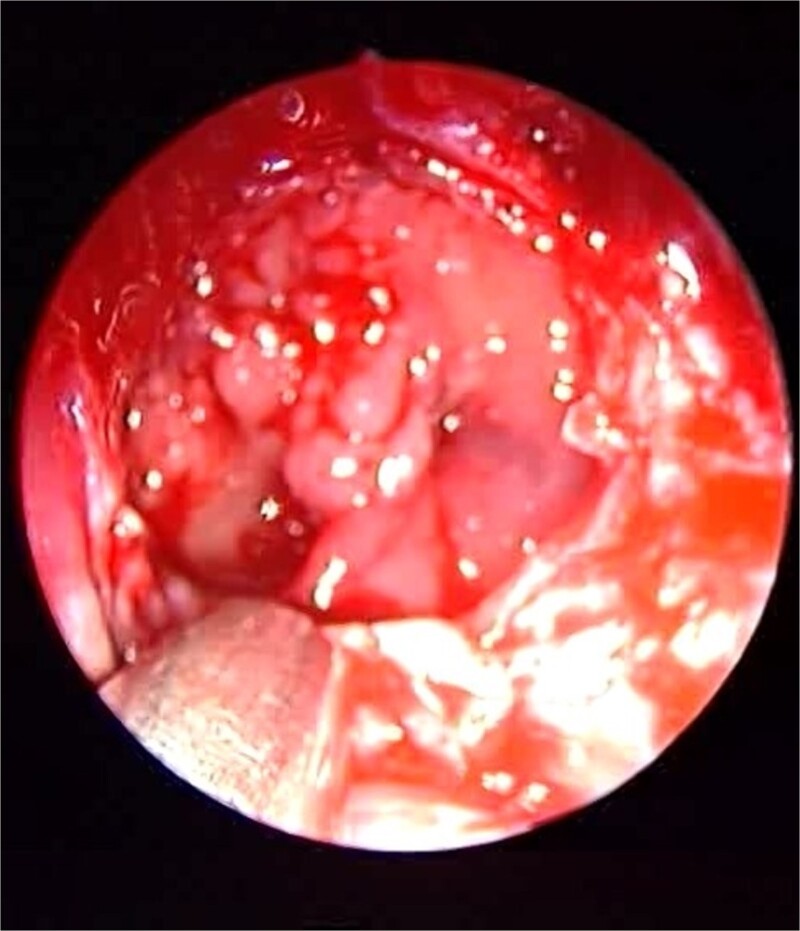
Endoscopic intraoperative image of the sphenoid sinus post removal of the fungal mud and mucin from the lateral wall of the sphenoid sinus.

Patient presented to the emergency department with four days history of sever left side headache associated with nausea and vomiting, and nasal discharge with on\off nasal obstruction. Two days history of diplopia with lateral gaze of the left eye, with decrease the visual acuity. Ophthalmologic examination revealed left abducens nerve palsy. Other neurologic examinations were within normal. Endoscopic nasal examination revealed left multiple grade two nasal polyps occupying the middle meatus. Pre-operative brain computed tomography (CT) shows bilateral near total opacification and expansion of the paranasal sinuses and nasal cavity (Fig. 1). Additionally, there is evidence of invasion of the left cavernous sinus with encasement of cavernous segment of left internal carotid artery (ICA). Furthermore, pituitary gland and optic chiasm displaced superiorly. Magnetic resonance imaging revealed that the sphenoid sinus is expanded and bulging superiorly into the pituitary fossa causing displacement of pituitary gland and stalk without mass effect upon the optic chiasm (Fig. 2). It extends laterally causing effacement of the cavernous sinus and left Meckel’s cave. No evidence of intracranial extension. The patient underwent functional endoscopic sinus surgery with computer-assisted navigation system. Intraoperatively, sphenoid sinus was full of fungal mud and mucin (Fig. 3). The polyps were removed completely from the nasal cavities, fungal mud, and mucin were removed from the sinuses (Figs 4 and 5). Patient had dramatic improvement after surgery, the rhinosinusitis symptoms are resolved, the diplopia has been improved, and the paranasal sinuses were clear. The patient was discharged with topical corticosteroid and saline irrigations.

## Discussion

The sixth cranial nerve (CN VI), the abducens, is entirely motor and extends from the brainstem to the lateral rectus. Passes via the Dorello’s canal in the company of the inferior petrosal sinus before entering the cavernous sinus to be known as the cavernous segment of the abducens nerve, then enters the orbit via the annulus of Zinn [5]. The cavernous sinus is a paired dural venous sinus located within the middle cranial fossa, on either side of the sella turcica of the sphenoid bone. Each cavernous sinus has a close anatomical relationship with several important vital structures. Abducens nerve, carotid plexus and ICA pass through the cavernous sinus to enter the orbit. Furthermore, oculomotor nerve (CN III), trochlear nerve (CN IV), ophthalmic (V1) and maxillary (V2) branches of the trigeminal nerve are passing through cavernous sinus lateral wall [6]. Due to its extended and convoluted course within the cranium, its dual connections to the base of the skull, as well as the adjacency of the cavernous sinus segment of the abducens nerve (CNVI) to the paranasal sinuses, the abducens nerve is susceptible to dysfunction arising from sinus-related diseases Including but not limited to fungal and bacterial infections [7]. There are two possible mechanisms of cranial neuropathies due to rhinosinusitis which are inflammation affecting adjacent nerves and mechanical compression that cause direct nerve compression and/or a circulatory disorder due to vascular compression [8]. Since (CN VI) supplies lateral rectus muscle which abducts the eye on the ipsilateral side, nerve palsy caused marked diplopia with limitation in lateral direction eye movement [9]. In clinical practice, the cause and degree of injury to the nerve and its vasculature have a significant impact on its recovery. As mentioned in the literature, patients with abducens nerve palsy secondary to allergic fungal sinusitis have good prognosis and postoperative resolution of symptoms [7, 10]. The treatment of AFRS is combination of surgical and medical approach. It consists of decompression of the sinus and relief of the expansion that compressing the orbit, brain, and nerve in addition to corticosteroid. Thereafter, adjuvant medical care includes oral and topical steroids are important in reducing the recurrence [11]. In the present case, FESS was performed within no more than 1 week from the onset of the ophthalmic symptoms. There was dramatic post-operative abducens nerve paralysis improvement.

## Conclusion

This case report demonstrates a unique presentation of abducens nerve palsy secondary to allergic fungal sinusitis which had dramatic improvement and resolution of the diplopia after the endoscopic sinus surgery. Steroids and nasal saline irrigation have been prescribed post operatively to prevent the recurrence.
